# The Moss *Physcomitrella patens* as a Model System to Study Interactions between Plants and Phytopathogenic Fungi and Oomycetes

**DOI:** 10.4061/2011/719873

**Published:** 2011-08-04

**Authors:** Inés Ponce de León

**Affiliations:** Departamento de Biología Molecular, Instituto de Investigaciones Biológicas Clemente Estable, Avenida Italia 3318, 11600 Montevideo, Uruguay

## Abstract

The moss *Physcomitrella patens* has a great potential as a model system to perform functional studies of plant interacting with microbial pathogens. *P. patens* is susceptible to fungal and oomycete infection, which colonize and multiply in plant tissues generating disease symptoms. In response to infection, *P. patens* activates defense mechanisms similar to those induced in flowering plants, including the accumulation of reactive oxygen species, cell death with hallmarks of programmed cell death, cell wall fortification, and induction of defense-related genes like *PAL*, *LOX*, *CHS*, and *PR-1*. Functional analysis of genes with possible roles in defense can be performed due to the high rate of homologous recombination present in this plant that enables targeted gene disruption. This paper reviews the current knowledge of defense responses activated in *P. patens* after pathogen assault and analyzes the advantages of using this plant to gain further insight into plant defense strategies.

## 1. Activation of Plant Defense Mechanisms against Pathogens

Over the last two decades, significant progress has been made on the mechanisms developed by flowering plants to ward off pathogen attack and the strategies used by plant pathogens to cause disease and manipulate host defense through secretion of virulence effector molecules [1–3]. Fungal and oomycetes pathogenicity factors have been isolated, and mechanisms utilized by the plant to recognize the pathogen and initiate a defense response have been identified. The effectiveness of this response relies on the ability to recognize the invading pathogen and to mount rapidly a host defense response that includes cell wall fortification, release of reactive oxygen species (ROS), induction of hypersensitive response (HR), and accumulation of phytoalexins and enzymes that degrade fungal cell walls, as well as other proteins involved in defense signaling [2, 4, 5]. Defense hormones such as salicylic acid (SA), jasmonates, and ethylene play key roles in regulating many of these host reactions to pathogen assault and modulation of additional hormonal pathways contribute to disease resistance [4, 6]. 

In contrast, in nonvascular plants like mosses (bryophytes), limited information is available on pathogen infection strategies as well as host defense mechanisms activated after microbial assault. Mosses are basal land plants that have diverged from flowering plants at least 450 million years ago after the colonization of land by an ancestor most closely related to modern green algae [7]. The transition of plants from water to land was accompanied by environmental adaptations related to terrestrial abiotic stresses, including a strengthened tolerance to radiation, extreme temperature, and drought [8]. As part of this transition to land, mosses have also strengthened defense strategies to cope with airborne pathogen attack and insect/animal predation. Since mosses are an evolutionary link between green algae and angiosperms, they can provide new insights into the evolution of plant defense against pathogenic microorganisms. The present review is focused on current knowledge related to the defense mechanisms activated in the moss *Physcomitrella patens* (*P. patens*) after fungal and oomycete infection and the advantages this plant possesses as a model system to study the interactions between plants and pathogens.

## 2. Interaction of Mosses with Fungal Pathogens in Nature

The presence of fungal pathogens in moss populations as well as the development of disease symptoms associated to fungal pathogenesis including chlorosis and necrosis was reported many decades ago [9–13]. In nature, the oomycete *Pythium ultimum* and the fungi *Thyronectria hyperantartica*, *Tephrocybe palustris*, *Bryoscyphus dicrani*, *Scleroconidioma sphagnicola*, *Acrospermum adeanum*, *Arrhenia retiruga*, *Lizonia baldinii,* and *Atradidymella muscivora* cause the formation of areas of dying and dead moss gametophytes [14, 15]. The fungal penetration process into moss tissues, as well as cell disruption and some host responses including the deposition of darkly pigmented material in the cell wall (papillae), was described for some of these pathogens [11, 14–16]. The penetration of host cells by bryophilous pathogens involves vegetative hyphae, penetration pegs and sometimes appressorium, and enzymatic digestion of the plant cell wall [13, 15].

## 3. Advantages of Using *P. patens* to Study Plant-Pathogen Interaction

**Figure 1:**
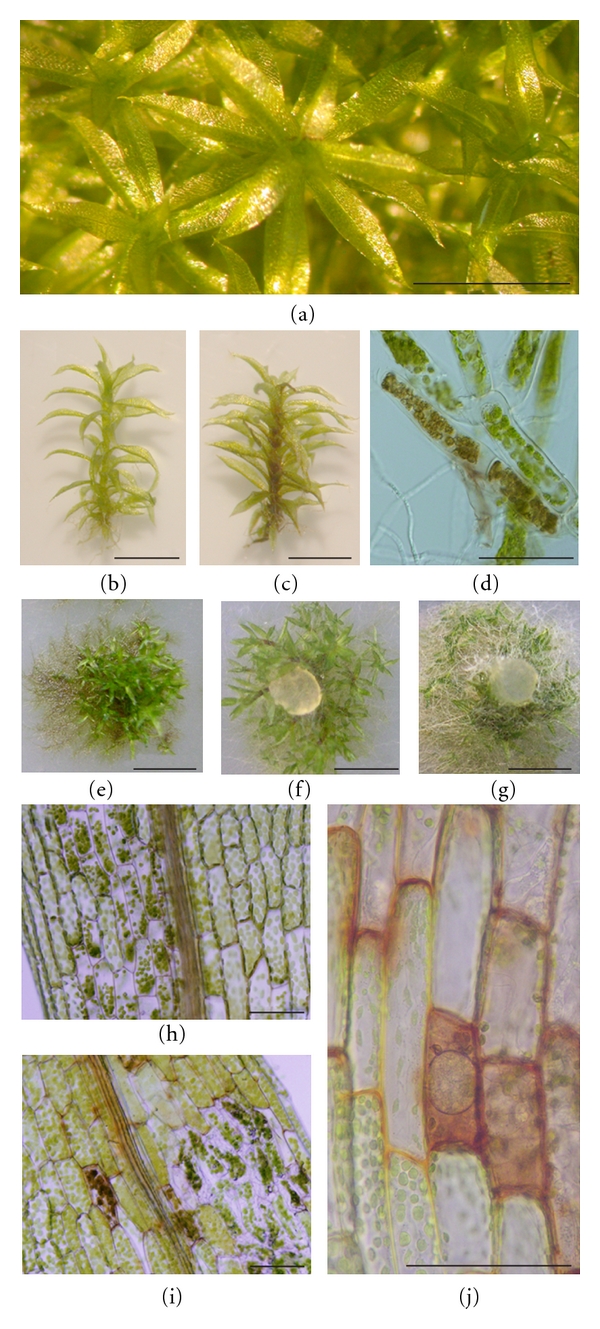
Disease symptoms in pathogen-infected moss tissues. (a) Healthy gametophytes, (b) Healthy gametophore, (c) *P. irregulare*-inoculated gametophore, (d) *B. cinerea*-inoculated protonema filaments, (e) *B. cinerea*-inoculated moss colony, (f) *P. irregulare*-inoculated colony, (g) *P. irregulare*-inoculated colony showing mycelium covering plant tissues, (h) *P. irregulare*-inoculated leaf, (i) *B. cinerea* inoculated leaf, (j) *P. debaryanum*-inoculated leaf showing an oospore. The *scale bar* represents 0.9 mm (a–c), 4 mm (e–g), and 20 *μ*m (d, h–j).

Recently, the moss *Physcomitrella patens* (*P. patens*) has emerged as a model plant to analyze plant interactions with microbial pathogens since it has several interesting features. *P. patens* has a relatively simple developmental pattern that resembles the basic organization of the body plan of flowering plants. This moss can be easily grown *in vitro,* and the susceptibility of distinct tissues to pathogens can be studied, since plants can be maintained as a haploid gametophyte with distinct developmental stages. The gametophyte consists of filamentous protonema (juvenile growth form) and gametophores (adult growth form) which are leafy shoots composed of a nonvascular stem with leaves and rhizoids (Figures 1(a)–1(e)) [17]. A further advantage is that leaves, rhizoids, and protonemal filaments consist of only one layer of cells making disease and host response assessment easily followed by microscopic analysis. In addition, *P. patens* is an excellent system to perform plant functional analysis of genes with possible roles in defense due to its high rate of homologous recombination, comparable to yeast cells, that enables targeted gene disruption [18]. Genomic resources for this moss are available and include ESTs and full-length cDNA collections (http://moss.nibb.ac.jp/), microarrays [19, 20], and the annotated genome (http://www.cosmoss.org/ and http://www.phytozome.net/) [8]. The presence of a dominant haploid gametophytic phase in *P. patens *allows the detection of mutant phenotypes in primary transformants, eliminating the need of backcrosses [21]. 

Lehtonen et al. have used *P. patens* to analyze the role of a class III peroxidase, by generation of a knockout line, in response to infection with two bryophilous fungi, a saprophytic isolate of the genus *Irpex* (Basidiomycota) and a pathogenic isolate of *Fusarium *sp. (Hyphomycetes), isolated from the moss *Racomitrium japonicum* grown in nature [22]. These authors could show that this peroxidase has an important role in protecting *P. patens* against invasion by saprophytes and fungal pathogens that are pathogenic on other mosses.

**Figure 2:**
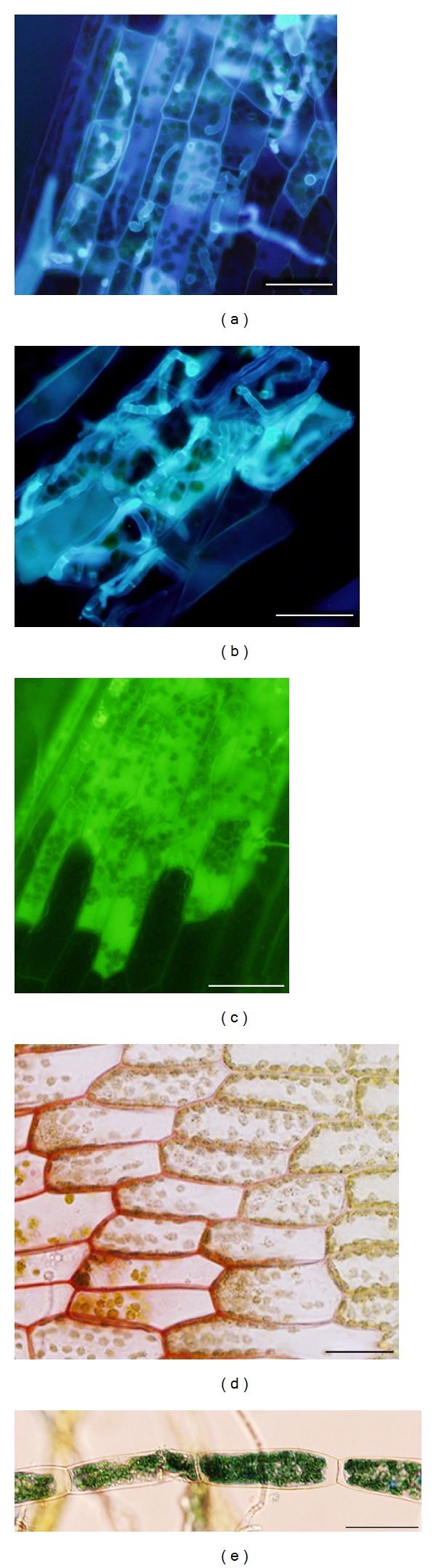
Pathogen growth and *P. patens* responses. *P. irregulare*-infected leaf (a) and *B. cinerea*-infected leaf (b) showing hyphal tissues stained with the fluorescent dye solophenyl flavine 7GFE 500, (c) ROS production in *P. debaryanum*-infected leaf stained with H_2_DCFDA, (d) Incorporation of phenolic compounds in cell walls of a *P. irregulare*-infected leaf visualized by safranin-O, (e) Evans blue staining of *B. cinerea*-inoculated protonemal filaments showing cytoplasmic shrinkage. The *scale bar* represents 20 *μ*m.

In addition to studying the interaction of *P. patens* with bryopathogenic fungi, *P. patens* can be used to understand in more detail how pathogens producing severe damage to important crops cause disease and how plants respond by activating a complex defense mechanism. Recently, we have shown that the broad host range ascomycete *Botrytis cinerea* (*B. cinerea*) and the oomycetes *Pythium irregulare *and *Pythium debaryanum* infect and multiply in *P. patens* gametophyte causing browning, necrosis, and maceration of the tissues [23, 24]. Necrosis and death of *P. patens* cells associated with *B. cinerea* and *Pythium* infection can be attributed to lytic enzymes and/or toxin production, since both *B. cinerea* and *Pythium* species are capable of producing a wide range of toxic metabolites and cell wall degrading enzymes facilitating tissue maceration [25–27]. Other fungal pathogens of crop plants, including *Verticillium dahlia*, *Aspergillus niger*, *Sclerotia sclerotorum* and *Fusarium graminearum,* also caused extensive cell death of *P. patens* tissues [28]. *B. cinerea *and *Pythium* mycelium grow within moss tissues, and hyphal tissues progress rapidly in dying and dead gametophytes leading finally to plant decay (Figures 1(g), 2(a)-2(b)). These necrotrophs are capable of infecting protonemal filaments, stems, rhizoids, and leaves, leading to browning of gametophytic tissues (Figures 1(b)–1(j)), [23, 24]. Penetration of moss cells by *B. cinerea *and *Pythium *involves a penetration peg or an appressorium, and hyphal tissues continue invading host tissues intracellularly and outside the cells in order to colonize new tissues (Figures 2(a)-2(b)). *P. patens* is fully susceptible to *B. cinerea* and *Pythium* infection, and in case of *Pythium* colonization, oospores were detected in moss-infected tissues within 2 days, indicating that the lifecycle was completed (Figure 1(j)), [24]. 

Several genomes of plant pathogens have been sequenced, including *B. cinerea* and *Pythium ultimum* (http://www.broadinstitute.org/annotation/genome/botrytis_cinerea.2/), [29], and specific genes associated with virulence and lifestyles have been identified. The strategies of infection and the role of the different effectors in promoting virulence and suppressing host defenses can be assessed in *P. patens*.

## 4. Defense Responses Activated in *P. patens* after Fungal and Oomycete Infection

Pathogens-produced effectors are recognized directly or indirectly by host resistance (R) genes of flowering plants leading to a resistance response known as effector-triggered immunity (ETI), which includes the localized programmed-cell-death (PCD-) HR, to restrict pathogen growth [2]. Probably, *P. patens* utilizes similar mechanisms for pathogen recognition since typical R genes are present in its genome [30]. Other defense responses similar to those activated in flowering plants after microbial pathogen infection are induced in *P. patens*, including the release of ROS and induction of programmed cell death (PCD), reinforcement of the cell wall, and activation of defense gene expression [23, 24, 28].

The release of ROS is an important plant defense response to pathogens involved in cell wall strengthening to restrict fungal or oomycete spread, intracellular signaling to activate further defense responses, induction of the HR, or by their direct toxicity to pathogens [31–33]. In *P. patens*, both *Pythium irregulare* and *Pythium debaryanum* [24] and *B. cinerea* (Ponce de León et al., unpublished results) cause an increase in ROS production (Figure 2(c)). Since these pathogens are necrotrophic pathogens, they can stimulate ROS production for their own advantage causing cellular damage and subsequent cell death [34]. *Pythium* and *B. cinerea* inoculation generates cell death and maceration of *P. patens* tissues, and in both cases, juvenile protonemal tissues showed higher maceration levels compared to gametophores [23, 24]. Similarly to what occurs in flowering plants,* B. cinerea*-inoculated tissues showed hallmarks of PCD including cytoplasmic shrinkage (Figure 2(e)), accumulation of autofluorescent compounds, and chloroplast breakdown [23]. Other features of PCD were observed in pathogen-infected *P. patens* tissues including nucleus condensation and DNA fragmentation, induction of nuclease activities, and formation of cytoplasmic vacuoles [28]. In addition, *P. patens* plants overexpressing the antiapoptotic gene Bax inhibitor-I showed resistance to necrotrophic fungal pathogens indicating that cell death in response to some pathogens is genetically programmed in mosses [28]. 

Modification of the plant cell wall is an important defense response against oomycetes and fungal pathogens. Bryophytes have thin cell walls where primary or secondary walls are not clearly distinguishable. Their cell walls contain cellulose, mannan, pectins, xyloglucan, and hydroxyproline-rich proteins, like flowering plants [35]. *P. patens* is a suitable plant system to analyze the role of plant cell wall during pathogen assault in a basal land plant since the occurrence of xyloglucans, rhamnogalacturonans, and hydroxyproline-rich proteins seem to have originated in bryophytes increasing cell wall strength compared with algae [35]. However, bryophytes do not possess lignins but instead have other phenolic compounds such as lignan or lignin-like polymers [36, 37]. *P. patens* response against *B. cinerea* and *Pythium* infection involves changes of the cell wall, including the accumulation of phenolic compounds (Figure 2(d)), and callose deposition in *Pythium*-infected leaves [23, 24]. In addition, we have observed enhanced expression of genes encoding hydroxyproline-rich proteins in *B. cinerea*-infected gametophytic tissues [Ponce de León et al. unpublished results], indicating that reinforcement of the moss cell wall is part of the defense mechanism activated against fungal pathogen.

In flowering plants, activation of defense responses following pathogen infection is associated with induction of a large number of host genes [38]. Some of the pathogen-induced genes encode enzymes involved in the synthesis of antimicrobial compounds, enzymes of oxidative stress protection, tissue repair, and cell wall reinforcement, whereas others encode proteins with regulatory functions in defense signaling pathways. *B. cinerea* and *Pythium*-infected *P. patens* plants showed enhanced expression of PAL (phenylalanine ammonia-lyase), CHS (chalcone synthase), LOX (lipoxygenase), and the classical marker of host defense response in flowering plants, PR-1 [23, 24]. These genes encode enzymes involved in the synthesis of phenylpropanoid, flavonoid, and oxylipins, respectively, with different roles in defense responses. Similarly to what happens in flowering plants, the products of these enzymes are likely to play a role in the defense response of *P. patens* against *B. cinerea* and *Pythium*. Infection of *P. patens* with fungal pathogens also induces the expression of genes associated with induction of PCD, like those encoding proteases, nucleases, and Bax Inhibitor-I involved in PCD regulation [28]. In addition, the expression of a gene encoding an alpha-Dioxygenase, involved in the protection of cell death caused by ROS and an avirulent *Pseudomonas syringae* strain in *Arabidopsis* [39], is rapidly induced in *P. patens* tissues after pathogen assault [40]. Since *P. patens* is an excellent model plant to perform functional studies by generation of targeted gene knockout lines, once pathogen-inducible moss genes have been identified, their contribution to disease resistance can be analyzed. *P. patens* has been shown to be a choice when only one or two copies of certain genes are present compared to large gene families in flowering plants. More clear-cut phenotypes have been observed in *P. patens* mutants compared to silenced or mutant lines of the homologous genes in *Arabidopsis* for Bax inhibitor-1 in response to biotic stress [28], and for a dehydrin protein in response to abiotic stress [41]. 

A high number of family members devoted to metabolism are present in the *P. patens *genome [8]. This moss possesses a higher number of members of the PAL and CHS multifamilies, as compared to flowering plants [42, 43], and some of them are induced after pathogen assault [23, 24]. While *P. patens* PAL gene family consists of 14 members and CHS family of 19 members, *Arabidopsis* has only 4 and one functional member, respectively [43]. In *P. patens*, novel metabolites can be generated by these enzymes with possible roles in defense against fungal and oomycete infection. Bryophytes are a rich source of secondary metabolites, and several flavonoids synthesized by CHS with antimicrobial activities have been identified [44]. Moreover, *P. patens* can use both C18-fatty acids and C20-fatty acids as precursors of oxylipins, while in flowering plants, oxylipins are mainly formed from C18-fatty acids [45, 46]. Thus, *P. patens* synthesizes typical plant oxylipins in addition to known oxylipins present in algae, animal, and mushrooms. 12-oxophytodienoic acid (cis-OPDA), which is the precursor of the defense hormone JA, was detected in healthy *P. patens* tissues with similar levels to those observed in *Arabidopsis* [47], and OPDA levels increased in this moss after *Pythium* infection [24]. However, the presence of JA in *P. patens* is still controversial. Stumpe et al. [47] could not detect any amount of JA or amino acid conjugates of JA in untreated moss tissues, while low levels of JA were detected in *Pythium*-inoculated *P. patens* gametophytes [24]. These differences can be due to the experimental conditions used in both studies, healthy versus pathogen-challenged tissues. Recently, the *B. cinerea* and *Pythium*-inducible *P. patens* LOX [23, 24], were shown to have a novel multifunctional activity with fatty acid hydroperoxide cleaving activity resulting in arachidonic acid- (a C20-fatty acid-) derived oxylipins not present in flowering plants [45, 46]. Volatile oxylipins derived from arachidonic acid were also detected after wounding *P. patens* tissues [48]. Novel acetylenic oxylipins have been identified in other mosses [49], and it remains an open question if some of these metabolites play a role in defense against fungal and oomycete pathogens. *P. patens* is for these reasons a very suitable system allowing the identification of new defense-related compounds that could have been changed or lost during the evolution of plants. From the 30.000 different protein-encoding *P. patens* genes, about 100 do not have clear homologues in flowering plants and can be considered novel moss genes [50]. Hence, 450 million years of evolution could have generated specific and novel defense mechanisms or strategies in mosses to cope with pathogens.

## 5. Conclusions

The use of *P. patens* as a plant system to analyze plant interactions with important fungal and oomycetes pathogens will provide valuable contributions to the biochemical and molecular mechanisms and components involved in plant defense. As an evolutionary link between green algae and angiosperms, *P. patens* allows an evolutionary analysis of plant defense during land colonization. In addition, *P. patens* offers interesting features compared to flowering plants, including the generation of knockout mutants and single-point mutations of genes involved in host resistance, the identification of clear mutant phenotypes due to a dominant gametophytic phase, and the identification of a diverse array of metabolites, some of which are not present in flowering plants and can play a role in defense responses. The onset of new-generation sequencing techniques together with functional studies will allow the identification of genes and products involved in plant resistance and may contribute to the development of novel and sustainable strategies to control disease in crop plants.
